# Seasonal Variation in Total Phenolic and Flavonoid Contents and DPPH Scavenging Activity of *Bellis perennis* L. Flowers

**DOI:** 10.3390/molecules15129450

**Published:** 2010-12-21

**Authors:** Tomáš Siatka, Marie Kašparová

**Affiliations:** Department of Pharmacognosy, Faculty of Pharmacy in Hradec Králové, Charles University in Prague, Heyrovského 1203, 500 05 Hradec Králové, Czech Republic

**Keywords:** *Bellis perennis*, flowers, phenolics, flavonoids, antioxidant activity

## Abstract

Variations in total phenolic and flavonoid contents as well as antioxidant activity of *Bellis perennis* (common daisy) flowers were investigated. The flowers were collected monthly (from March to October, *i.e.*, during the usual flowering season of the plant) at three localities in three different years. Total flavonoids were determined spectrophotometrically by two methods: by formation of a complex with aluminium chloride after acidic hydrolysis of flower extracts (method 1) and by reaction with boric and oxalic acids in extracts without their modification (method 2). Total phenolics were determined spectrophotometrically using the Folin-Ciocalteu reagent. The antioxidant activity was determined spectrophotometrically by a 1,1-diphenyl-2-picrylhydrazyl (DPPH) radical scavenging assay. The contents of flavonoids varied from 0.31 to 0.44 mg quercetin equivalent/100 mg dry weight (method 1) and from 1.37 to 2.20 mg apigenin-7-glucoside equivalent/100 mg dry weight (method 2). Total phenolics ranged from 2.81 to 3.57 mg gallic acid equivalent/100 mg dry weight. The antioxidant activity expressed as IC_50_ values varied from 66.03 to 89.27 µg/mL; it is about 50, 30, 20, and 10 times lower as compared with quercetin, ascorbic acid, Trolox^®^, and butylhydroxytoluene, respectively, and about five times higher in comparison with apigenin-7-glucoside. There is a significant correlation between antioxidant activity and total phenolics. No correlation between total flavonoid contents and antioxidant activity was observed. Contents of phenolics and flavonoids as well as antioxidant activity of daisy flowers vary to a relatively small extent during the year and are not dependant on the time of collection. Thus, the flowers possess comparable quality as to these characteristics over the whole flowering season of *Bellis perennis*. Effects of environmental factors on the amounts of secondary metabolites in plants are also discussed.

## 1. Introduction

*Bellis perennis* L., the common daisy or English daisy, is a small perennial herb belonging to the family *Asteraceae* (*Compositae*). It is native to Europe and western Asia [1,2], and introduced in North America, South America, and New Zealand [3]. The species has a very long flowering season, with flowers being produced mainly from about March to November [1,2]. The common daisy is used in the traditional medicine as an expectorant [4,5], a diuretic [5,6], an anti-inflammatory [4,5,7], and a vulnerary [8]. The flowers and young leaves are used as a vegetable [9].

The phenolic constituents of *Bellis perennis* include flavonoids [5,11,12,13], anthocyanins (three glucuronylated and malonylated cyanidin-3-glucosides) [13], tannins [14], and phenolic acids (e.g., caffeic, ferulic, sinapic, p-coumaric, and salicylic acids) [7]. The following flavonoids were described in daisy flowers: quercetin, apigenin, kaempferol [10], isorhamnetin [5], apigenin-7-O-β-D-glucoside, apigenin-7-O-β-D-glucuronide [12], apigenin-7-O-(6´´-*E*-caffeoyl)-β-D-glucoside, apigenin-7-O-β-D-methylglucuronide [10], isorhamnetin-3-O-β-D-galactoside, isorhamnetin-3-O-β-D-(6´´-acetyl)-galactoside, and kaempferol-3-O-β-D-glucoside [11]. The contents of flavonoids after acid hydrolysis were determined spectrophotometrically according to Christ and Müller (0.25% or 1.32%, expressed as quercetin or apigenin, respectively) and by HPLC (sum of flavonoids: 0.23%; derivatives of quercetin, apigenin, kaempferol, and isorhamnetin: 0.013%, 0.10%, 0.007%, and 0.019%, respectively) [5]. *Bellis perennis* also contains triterpenoid saponins [9,15], polyacetylenes [16,17], and essential oil [8]. The haemolytic activity, which was used for quantification of saponins in daisy flowers, changes considerably during the year; it is the lowest in March, then it increases gradually, reaching a maximum in summer months (June, July, and August; four- to six-fold values as compared with March), and then it decreases again [18].

Flavonoids belong to a large group of plant-derived low-molecular-weight polyphenolic compounds biosynthesized from both the shikimic acid and acetic acid pathways [19]. They deserve attention because of their biological properties, such as vascular protective and venous tonic, anti-inflammatory, diuretic, antispasmodic, hepatoprotective, and hypocholesterolemic effects [19,20]. There is a lot of interest in the antioxidant activity of flavonoids and other plant phenolic compounds due to their potential in health promotion and disease prevention [21,22,23,24]. Antioxidants are able to scavenge free radicals by various mechanisms [23,24], and thus to contribute to protection of biologically important cellular components, such as DNA, proteins, and membrane lipids, from free radical attacks leading to cell damage, which has been linked to aging, inflammation, atherosclerosis, ischemic injury, and cancer [19,22,25].

There is a little information about variation in quantities of phenolic constituents in daisy flowers during the year. The aim of this work was to investigate changes in total phenolic and flavonoid contents and DPPH radical scavenging activity of *Bellis perennis* flowers collected over the flowering period.

## 2. Results and Discussion

Flowers of *Bellis perennis* were gathered monthly during daisy's usual flowering period at three places of the Czech Republic (Ústí nad Labem, Dobré, and Hradec Králové) in three different years (2000, 2001, and 2003) in order to obtain randomly varying material as for locality and environmental conditions. There were no morphological differences among *Bellis perennis* plants from individual localities.

Total flavonoids in daisy flowers were determined spectrophotometrically by the two pharmacopoeial methods: according to Christ and Müller by formation of a complex with aluminium chloride after acidic hydrolysis of extracts (the contents of flavonoids were expressed as quercetin equivalents on dry weight) [26,27] (method 1), and according to Glasl by reaction with boric and oxalic acids in extracts without their modification (the contents of flavonoids were expressed as apigenin-7-glucoside equivalents on dry weight) [28,29] (method 2). The results are given in Table 1. The contents of flavonoids determined by method 1 are 0.31–0.42, 0.31–0.44, and 0.34–0.43 mg/100 mg, and the maximal content is 1.35, 1.42, and 1.26 times higher than the minimal one in flowers from Ústí nad Labem, Dobré, and Hradec Králové, respectively. The flavonoid contents determined by method 2 vary from 1.47 to 1.98, 1.69 to 2.20, and 1.37 to 2.07 mg/100 mg, and the highest flavonoid level is 1.34, 1.30, and 1.51-fold higher than the lowest one in flowers from Ústí nad Labem, Dobré, and Hradec Králové, respectively. Thus, the inter-monthly differences of total flavonoid contents are relatively small. No common maximum or minimum in flavonoid amounts (no matter whether determined by method 1 or 2) connected with a certain part of the year was observed. No correlation between total flavonoid contents measured by the two methods was found. It can be explained by different sensitivity of both methods to individual compounds which may vary in their amounts and proportions in daisy flowers during the year, so the total contents change to a different extent and do not correlate.

Total phenolic contents were determined spectrophotometrically by the most common method of Singleton and Rossi using the Folin-Ciocalteu reagent and expressed as gallic acid equivalents [30]. The results are shown in Table 1. The contents of total phenolics vary from 2.94 to 3.41, 3.03 to 3.52, and 2.81 to 3.57 mg/100 mg, and the maximal value is 1.16, 1.16, and 1.27 times higher than the minimal one in flowers from Ústí nad Labem, Dobré, and Hradec Králové, respectively. There is no correlation between the total determined content of phenolic and flavonoid compounds. It should be pointed out that some non-phenolic reducing compounds, such as sugars [30,31], organic acids [32,33], ascorbic acid [30,33], and amino acids [31,34], could interfere with the determination of total phenolics in the Folin-Ciocalteu assay and lead to an overvaluation of the phenolic content [35]. Furthermore, different phenolics present different responses with the Folin-Ciocalteu reagent, for example, while gallic acid, rutin and caffeic acid have a similar behaviour, several flavonoids exhibit low absorption, which leads to an underestimation of various compounds [32,36].

**Table 1:** Contents of total flavonoids and total phenolics, and DPPH scavenging activity of *Bellis perennis* flowers.

Locality Month of collection	Total flavonoids (method 1) mg/100 mg dw	Total flavonoids (method 2) mg/100 mg dw	Total phenolics mg/100 mg dw	DPPH scavenging activity (IC_50_) µg/mL
**Ústí nad Labem**				
March	0.35 ± 0.01	1.76 ± 0.06	3.10 ± 0.04	74.83 ± 1.73
April	0.42 ± 0.01	1.98 ± 0.03	3.17 ± 0.02	78.18 ± 0.71
May	0.42 ± 0.01	1.70 ± 0.09	3.41 ± 0.05	71.42 ± 2.43
June	0.39 ± 0.01	1.63 ± 0.02	3.14 ± 0.05	77.49 ± 2.13
July	0.37 ± 0.01	1.68 ± 0.02	3.25 ± 0.13	75.02 ± 2.06
August	0.40 ± 0.02	1.47 ± 0.03	2.94 ± 0.01	83.37 ± 3.44
September	0.31 ± 0.01	1.84 ± 0.10	3.03 ± 0.02	83.12 ± 0.91
October	0.39 ± 0.02	1.56 ± 0.05	3.01 ± 0.02	74.67 ± 4.26
**Dobré**				
March	0.31 ± 0.01	1.69 ± 0.06	3.19 ± 0.05	78.13 ± 3.53
April	0.43 ± 0.03	2.17 ± 0.05	3.46 ± 0.05	74.67 ± 8.39
May	0.43 ± 0.01	1.80 ± 0.04	3.44 ± 0.10	78.28 ± 0.36
June	0.39 ± 0.01	2.20 ± 0.16	3.52 ± 0.01	66.47 ± 1.57
July	0.39 ± 0.01	1.77 ± 0.01	3.03 ± 0.01	89.27 ± 7.28
August	0.44 ± 0.01	2.00 ± 0.01	3.30 ± 0.02	87.74 ± 1.77
September	0.38 ± 0.01	1.92 ± 0.10	3.24 ± 0.02	76.97 ± 1.11
October	0.43 ± 0.01	1.72 ± 0.01	3.03 ± 0.04	81.71 ± 8.35
**Hradec Králové**				
April	0.37 ± 0.02	1.88 ± 0.05	3.53 ± 0.07	73.16 ± 1.65
May	0.35 ± 0.02	1.90 ± 0.05	3.57 ± 0.02	66.03 ± 1.05
June	0.35 ± 0.01	1.37 ± 0.01	3.03 ± 0.11	79.20 ± 0.15
July	0.34 ± 0.01	1.42 ± 0.05	2.92 ± 0.16	83.95 ± 0.01
August	0.37 ± 0.01	1.46 ± 0.01	2.81 ± 0.12	82.14 ± 0.62
September	0.37 ± 0.01	2.07 ± 0.11	3.40 ± 0.13	71.77 ± 1.95
October	0.43 ± 0.01	1.96 ± 0.08	3.12 ± 0.16	80.73 ± 0.76

Results are means ± SD of three different samples.

The antioxidant activity was determined by a widely used and convenient method – the DPPH radical scavenging assay [37,38]. The radical scavenging activity of flower extracts was expressed as the IC_50_ value (µg/mL), *i.e.*, the concentration necessary to decrease the DPPH concentration by 50%. As shown in Table 1, scavenging activities against DPPH radical are 71.42–83.37, 66.47–89.27, and 66.03–83.95 µg/mL, and the highest scavenging activity is 1.17, 1.34, and 1.27-fold higher than the lowest value in flowers from Ústí nad Labem, Dobré, and Hradec Králové, respectively. There is a statistically significant correlation between radical scavenging activity and total phenolics; correlation coefficients are 0.733, 0.707, and 0.927 for flowers from Ústí nad Labem, Dobré, and Hradec Králové, respectively. Total flavonoid contents and DPPH scavenging activity do not correlate, so that only part of the flavonoids of daisy flowers participates in the antioxidant activity. The radical scavenging activity of daisy flowers was compared with that of some reference compounds (Table 2); it is about 50, 30, 20, and 10 times lower as compared with quercetin, ascorbic acid, Trolox^®^, and butylhydroxytoluene, respectively, and approximately 5 times higher in comparison with apigenin-7-glucoside.

**Table 2:** DPPH scavenging activity of reference compounds.

Compound	DPPH scavenging activity (IC_50_) µg/mL
Quercetin	1.45 ± 0.03
Apigenin-7-glucoside	387.26 ± 2.91
Ascorbic acid	2.62 ± 0.02
Trolox^®^	3.67 ± 0.02
Butylhydroxytoluene	7.12 ± 0.32

Only small differences among total flavonoid and phenolic amounts and DPPH radical scavenging activity in flowers of *Bellis perennis* from three investigated localities were observed (Table 1). As to variations during the year, the total phenolic and flavonoid contents as well as DPPH radical scavenging activity of daisy flowers vary, but, in contrast to haemolytic activity [18], the changes are relatively small (Table 1). In addition, no general regularity in these changes, related to some months of the year, occurs. Statistical analysis of inter-monthly differences during the year at the three localities is given in Table 3. As shown in Table, a lot of differences are not significant or significant only in one or two years. In a few cases, the inter-monthly differences are significant in all three years (total flavonoids by method 1: September–October; total flavonoids by method 2: April–July and August; total phenolics: April–August, June and August–September; and DPPH scavenging activity: June–August). The changes of determined metabolites and antioxidant activity in daisy flowers seem to be brought about by immediate fluctuations in environmental factors, such as day and night temperatures, rainfalls, drought, and the duration and intensity of sunshine. The fluctuations vary among years and may explain irregularity of variations in investigated characteristics of daisy flowers. The influence of environmental conditions on quantity of active constituents in various plants has been reported. Phenolic acid, flavonol, and anthocyanin contents and antioxidant activity in fruit juice of *Fragaria* x *ananassa* were considerably influenced by different day/night growing temperature combinations [39]. Cool temperatures and irrigation have been shown to increase accumulation of isoflavones in seeds of *Glycine max* [40]. A positive correlation between the duration of exposure to sunshine and flavonoid content in leaves of *Angelica keiskei* has been demonstrated [41]. Assimilation rate and stomatal conductance were greater in leaves of *Olea europaea* plants grown under full-sun than under partial shading; concentration of quercetin and luteolin glycosides, but not that of apigenin glycosides increased in leaves fully exposed to sunlight irradiance in comparison with those under partial shading [42]. The effects of UV-A and UV-B irradiance in *Lactuca sativa* (red lettuce) were evaluated; plant growth (total above ground dry weight) was reduced, and accumulation of total phenolics, anthocyanins, and flavonoids in leaves was stimulated proportionally to the degree of ultraviolet radiation cutoff [43]. Physiological and growth responses of *Bellis perennis* to UV-A and UV-B irradiances [44,45], and temperature [46] have been described. Ultraviolet light influences dry weight of flowers, leaves, and roots, leaf area, photosynthetic parameters, and transpiration rate depending on wavelength and intensity of radiation [44,45], and temperature affects plant total dry mass, leaf area, and root respiration [46], so secondary metabolism in *Bellis perennis* may be influenced as well. Thus, variations in total phenolic and flavonoid contents and DPPH scavenging activity of daisy flowers reported in the present study can be explained by qualitative and quantitative changes of environmental circumstances during the year. It is difficult to determine which environmental factor is mainly responsible for observed variations because the changes are small and irregular. Moreover, the flowers were collected from plants growing in natural conditions, so it is not easy to separate effects of individual factors from multifactorial influence of the environment. This would be possible in greenhouse or laboratory experiments where the factors can be set and regulated exactly.

**Table 3:** Statistical analysis of inter-monthly differences in contents of total flavonoids and total phenolics, and DPPH scavenging activity of *Bellis perennis* flowers collected during the year at three localities (s – differences statistically significant, n – differences statistically not significant; *P* < 0.05).

		Locality, year																				
		Ústí nad Labem, 2000							Dobré, 2001							Hradec Králové, 2003						
	M^a^	3	4	5	6	7	8	9	3	4	5	6	7	8	9	4	5	6	7	8	9
**TF 1^b^**	**4**	s							s												
	**5**	s	n						s	n						n					
	**6**	s	s	s					s	n	s					n	n				
	**7**	n	s	s	n				s	n	s	n				n	n	n			
	**8**	s	n	n	n	n			s	n	n	s	s			n	n	n	s		
	**9**	s	s	s	s	s	s		s	n	s	n	n	s		n	n	n	s	n	
	**10**	s	n	n	n	n	n	s	s	n	n	s	s	n	s	s	s	s	s	s	s
**TF 2^c^**	**4**	s							s												
	**5**	n	s						n	s						n					
	**6**	s	s	n					s	n	s					s	s				
	**7**	n	s	n	s				n	s	n	s				s	s	n			
	**8**	s	s	s	s	s			s	s	s	n	s			s	n	n	n		
	**9**	n	n	n	n	n	s		s	s	n	n	n	n		n	n	s	s	s	
	**10**	s	s	n	n	s	n	s	n	s	s	s	s	s	n	n	n	s	s	s	n
**TP^d^**	**4**	n							s												
	**5**	s	s						s	n						n					
	**6**	n	n	s					s	n	n					s	s				
	**7**	n	n	n	n				s	s	s	s				s	s	n			
	**8**	s	s	s	s	n			s	s	n	s	s			s	s	n	n		
	**9**	n	s	s	s	n	s		s	s	n	s	s	s		n	n	s	s	s	
	**10**	s	s	s	s	n	s	n	s	s	s	s	n	s	s	s	s	n	n	n	n
**DSA^e^**	**4**	s							n												
	**5**	n	s						n	n						s					
	**6**	n	n	s					s	n	n					s	s				
	**7**	n	n	n	n				n	n	n	s				s	s	s			
	**8**	s	n	s	n	s			s	n	s	s	n			s	s	s	s		
	**9**	s	s	s	s	s	n		n	n	n	s	n	s		n	s	s	s	s	
	**10**	n	n	n	n	n	n	n	n	n	n	n	n	n	n	s	s	n	s	n	s

^a^M – month of collection: 3 – March, 4 – April, 5 – May, 6 – June, 7 – July, 8 – August, 9 – September, 10 – October; ^b^TF 1 – total flavonoids (method 1); ^c^TF 2 – total flavonoids (method 2); ^d^TP – total phenolics; ^e^DSA – DPPH scavenging activity

## 3. Experimental

### 3.1. Chemicals and analytical instruments

Reagents and solvents of analytical grade were purchased from Lachema (Brno, Czech Republic). 1,1-diphenyl-2-picrylhydrazyl, gallic acid, quercetin, apigenin-7-glucoside, ascorbic acid, Trolox^®^, and butylhydroxytoluene were purchased from Sigma-Aldrich (Prague, Czech Republic). An A 200 S analytical balance (Sartorius, Göttingen, Germany), a GFL 1042 water bath (Gesellschaft für Labortechnik, Burgwedel, Germany), a Laborota 4010 rotary evaporator (Heidolph Instruments, Schwabach, Germany), a U15C thermostat (Prüfgeräte-Werk, Medingen, Germany), and a 901 spectrophotometer (Büchi Labortechnik, Flawil, Switzerland) were used.

### 3.2. Plant material

Flower heads of *Bellis perennis* were collected at three localities of the Czech Republic (Ústí nad Labem, Dobré, and Hradec Králové; altitudes: 136 m, 440 m, and 220 m, respectively; soil types according to the World Reference Base for Soil Resources were: Haplic Luvisols from loamy loess, Haplic Cambisols from weathering products of subsilicic metasediment (greenschist), and Haplic Fluvisols from alluvial sediments, resp.; climate: temperate continental; additional climatic characterization of the localities is given in Table 4.) on one day between the 18th and 20th of every month from March to October in three different years (2000, 2001, and 2003, resp.; excepting March at Hradec Králové – daisies did not flower yet because of the snow and cold). The plants grew in a lawn. Species with known allelopathic potential were not present. The flower heads were air-dried in the shade at room temperature. Whole dried flower heads were stored in air-tight containers protected from light at room temperature. Plant materials were powdered before analyses and analysed together at the end of a particular year.

**Table 4:** Climatic characterization of localities (annual values).

	Locality, year		
	Ústí nad Labem, 2000	Dobré, 2001	Hradec Králové, 2003
Mean air temperature	11.1 °C	7.7 °C	10.4 °C
Total precipitation	574 mm	872 mm	581 mm
Sunshine duration	1434 h	1401 h	1776 h

### 3.3. Determination of total flavonoids

The determination of total flavonoids was performed spectrophotometrically by two pharmacopoeial methods [27,29]: according to Christ and Müller [26] (method 1), and Glasl [28] (method 2).

Method 1. *Stock solution*. Powdered plant material (0.600 g) was mixed with acetone (20 mL), aqueous solution of hexamethylenetetramine (1 mL, 5 g/L), and hydrochloric acid (2 mL, 25%). The mixture was boiled in a water bath under reflux for 30 min. The obtained extract was filtered, and the residue was extracted twice with acetone (2 × 20 mL) in a water bath under reflux for 10 min. The extracts were combined and diluted to 100 mL with acetone. A portion of this solution (20 mL) was diluted with distilled water (20 mL) and extracted once and then three times with ethyl acetate (15 mL and 3 × 10 mL, resp.). The ethyl acetate extracts were combined, washed twice with distilled water (2 × 50 mL), filtered over anhydrous sodium sulphate (10 g), and diluted to 50 mL with ethyl acetate. *Test solution*. The stock solution (10 mL) was mixed with a 2% (w/v) aluminium chloride solution in a 5% (v/v) solution of glacial acetic acid in methanol (1 mL) and diluted to 25 mL with a 5% (v/v) solution of glacial acetic acid in methanol. *Compensation solution*. The stock solution (10 mL) was diluted to 25 mL with a 5% (v/v) solution of glacial acetic acid in methanol. After 30 min, the absorbance of the test solution was measured at 425 nm, by comparison with the compensation solution. The content of total flavonoids was calculated using a calibration curve of quercetin (the linearity range: 0.1–20 µg/mL, R^2^ = 0.9999) and expressed as mg of quercetin equivalents per 100 mg of dry weight. All determinations were carried out in triplicate.

Method 2. *Stock solution*. Powdered plant material (0.400 g) was extracted with 60% (v/v) ethanol (40 mL) by shaking in a water bath at 60 °C for 10 min. The obtained extract was filtered, and the residue was extracted with 60% (v/v) ethanol (40 mL) by shaking in a water bath at 60 °C for 10 min. The extraction flask and the residue of plant material were rinsed with further 60% (v/v) ethanol, and the combined extracts were diluted to 100 mL with 60% (v/v) ethanol and filtered. *Test solution*. The stock solution (5 mL) was evaporated to dryness under reduced pressure. The residue was dissolved in a mixture of 10 volumes of methanol and 100 volumes of glacial acetic acid (11 mL). Then, a solution (10 mL) containing 25 g/L of boric acid and 20 g/L oxalic acid in anhydrous formic acid (98%) was added, and the volume was made up to 25 mL with anhydrous acetic acid (99.6%). *Compensation solution*. The stock solution (5 mL) was evaporated to dryness under reduced pressure. The residue was dissolved in a mixture of 10 volumes of methanol and 100 volumes of glacial acetic acid (11 mL). Then, anhydrous formic acid (98%, 10 mL) was added, and the volume was made up to 25 mL with anhydrous acetic acid (99.6%). After 30 min, the absorbance of the test solution was measured at 405 nm, by comparison with the compensation solution. The content of total flavonoids was calculated using a calibration curve of apigenin-7-glucoside (the linearity range: 0.8–24 µg/mL, R^2^ = 0.9999) and expressed as mg of apigenin-7-glucoside equivalents per 100 mg of dry weight of plant material. All determinations were carried out in triplicate.

### 3.4. Determination of total phenolics

Total phenolic contents were determined spectrophotometrically according to the method of Singleton and Rossi [30]. The stock solution from determination of total flavonoids (method 2) (2.5 mL) was diluted to 25 mL with distilled water. An aliquot of the solution (2 mL) was mixed with Folin-Ciocalteu reagent (10-fold diluted with distilled water, 10 mL). After 5 min, a 7.5% (w/v) sodium carbonate solution (8 mL) was added. After 2 hours, the absorbance was measured at 765 nm against a blank prepared as described above with distilled water (2 mL), Folin-Ciocalteu reagent, and sodium carbonate solution. The content of total phenolics was calculated using a calibration curve of gallic acid (the linearity range: 1–5 µg/mL, R^2^ = 0.9997) and expressed as mg of gallic acid equivalents per 100 mg of dry weight of plant material. All determinations were carried out in triplicate.

### 3.5. DPPH radical scavenging activity

The free radical scavenging activity was determined spectrophotometrically, using a stable free radical 1,1-diphenyl-2-picrylhydrazyl (DPPH) [38,47,48]. For each determination, the stock solution from determination of total flavonoids (method 2) was diluted to a dilution series (six different concentrations) with 60% (v/v) ethanol. An aliquot of each dilution (0.5 mL) was mixed with methanolic solution of DPPH (5 mL, 0.06 mM). The mixtures were shaken vigorously and incubated at 37 °C in the dark for 30 min. At the same time, a control containing 60% (v/v) ethanol (0.5 mL) and methanolic solution of DPPH (5 mL, 0.06 mM) was run. The absorbance was measured at 517 nm against methanol as a blank. The percentage of DPPH scavenging was calculated as follows: [(*A_control_* − *A_sample_*)/*A_control_*] × 100. The percentage of DPPH scavenging *versus* concentration of samples was plotted. The concentration of the sample necessary to decrease the DPPH concentration by 50% was obtained by interpolation from linear regression analysis and denoted IC_50_ value (µg/mL). All determinations were carried out in triplicate.

Quercetin, apigenin-7-glucoside, ascorbic acid, Trolox^®^, and butylhydroxytoluene were used as reference compounds. The tested compounds were dissolved in 60% (v/v) ethanol, and determination and calculation of DPPH radical scavenging activity was performed as described above.

### 3.6. Statistical analysis

Three different samples of every month were analysed, each one in triplicate. All values are means ± standard deviation of three samples. Statistical analysis was performed using a one-way analysis of variance (ANOVA), followed by Tukey’s multiple comparison test. Differences at *P* < 0.05 were considered statistically significant. Correlation between total phenolic content and antioxidant activity was established by linear regression analysis at *P* < 0.05.

## 4. Conclusions

Contents of phenolics and flavonoids as well as radical scavenging activity of daisy flowers vary to a relatively small extent during the year and are not dependant on the time of collection. In other words, the flowers may be collected from spring to autumn and are comparable in biological effects, for which phenolics and flavonoids are responsible, over the whole flowering season of *Bellis perennis*.
